# *Ancyronyx* Erichson, 1847 (Coleoptera, Elmidae) from Mindoro, Philippines, with description of the larvae and two new species using DNA sequences for the assignment of the developmental stages

**DOI:** 10.3897/zookeys.321.5395

**Published:** 2013-08-06

**Authors:** Hendrik Freitag

**Affiliations:** 1 Ateneo de Manila University, Department of Biology, School of Science & Engineering, Loyola Heights, Quezon City 1101, the Philippines; 2 Research Associate of Senckenberg Naturhistorische Sammlungen Dresden, Königsbrücker Landstrasse 159, D-01109 Dresden, Germany

**Keywords:** *Ancyronyx*, Spider Water Beetles, Elmidae, Coleoptera, taxonomy, new species, larva, DNAbarcoding, *cox1*, *cob*, Buhid, Mindoro, Philippines

## Abstract

*Ancyronyx buhid* **sp. n.** and *Ancyronyx tamaraw* **sp. n.** are described based on adults and larvae, matched using their *cox1* or *cob* DNA sequence data. Additional records of *Ancyronyx schillhammeri* Jäch, 1994 and *Ancyronyx minerva* Freitag & Jäch, 2007 from Mindoro are listed. The previously unknown larva of *Ancyronyx schillhammeri* is also described here, aided by *cox1* data. The new species and larval stages are described in detail and illustrated by SEM and stacked microscopic images. Keys to the adult and larval *Ancyronyx* species of Mindoro and an updated checklist of Philippine *Ancyronyx* species are provided. The usefulness as bioindicators, the phylogenetic relationships and biogeographic aspects affecting the distribution patterns are briefly discussed.

## Introduction

The so-called spider water beetles of the genus *Ancyronyx* Erichson, 1847, family Elmidae Curtis, 1830 are known from North America, China, South and Southeast Asia (Freitag 2012). The genus diagnosis was recently revised (Freitag 2012) due to the description of several new species in the last decade.

The genus appears closely related to *Podelmis* Hinton, 1941 but can be distinguish by the complete absence of a prosternal anterior process, and the shape of the terminal segment of the ovipositor (stylus slender and more or less straight versus somewhat conical and distinctly bent laterad in *Podelmis*). Two morphologically distinct groups of *Ancyronyx* are recognised, both are characterised by varying morphological and ecological adaptation patterns. The SE Asian representatives of the *Ancyronyx variegatus* group (sensu Freitag and Jäch 2007) of slightly larger species can be found in mesosaprobic rivers. The relatively smaller species of the *Ancyronyx patrolus* group (sensu Freitag and Jäch 2007) occur predominantly in clean permanent streams (Freitag 2012). When identified on species level, they might therefore serve as good bioindicators, just as Elmidae in general (e.g. Moog and Jäch 2003, Hilsenhoff 1982). As larvae of the respective taxa are naturally more abundant then adults, appropriate identification tools for these developmental stages might be of great interest for ecological and applied limnological studies. Larval stages of *Ancyronyx* have been formally described only from the Philippines so far (Freitag and Balke 2011), but the larval habitus of the only North American species was figured by Brown (1972).

This study is part of the Baroc River Catchment Survey of the Ateneo de Manila University which focuses on the Key Biodiversity Area “69 Hinunduang Mt.” (*sensu* Ong et al. 2002), classified as a terrestrial and inland water area of very high biological importance and extremely high critical conservation priority (“EHc”), under high socioeconomic pressure (Ong et al. 2002), which is, however, only subjected to moderate conservation efforts and not yet formally protected (Ambal et al. 2012).

Furthermore, previously collected specimens from Mindoro were included, that are partly from other conservation and research priority areas of high and highest urgency (“64 Naujan Lake National Park” and “62 Puerto Galera” *sensu* Ong et al. 2002).

## Material and methods

### Taxon Sampling

The material was preserved in 95% ethyl alcohol to allow genetic sequencing. Most material was retrieved during the ongoing Baroc River Catchment Survey. Material collections from the 1990s were examined at the Naturhistorisches Museum Wien, Austria(NMW), the Senckenberg Museum für Tierkunde Dresden, Germany(SMTD), and the Zoological Museum of the University Copenhagen, Denmark(ZMUC).

All specimens recorded by the first author were manually collected as indicated by letter “M” at the end of a collection label. Letter codes in parenthesis refer to a particular sampling station and microhabitat of the Baroc River Catchment. Number codes are arbitrary. They do not follow temporal or spatial patterns.

### DNA extraction and sequencing

DNA was extracted from five larvae and three adults (entire specimens) from Mindoro, and one entire adult specimen of the recently described *Ancyronyx jaechi* Freitag, 2012 from Sri Lanka using Qiagen DNeasy kit (Qiagen, Hilden, Germany). The extraction was done by a single elution following the protocol for animal tissues (Qiagen 2002). The 3’ end of the cytochrome *c* oxidase subunit I (*cox1*) gene was amplified using polymerase chain reaction(PCR) following standard protocols (see http://zsm-entomology.de/wiki/The_Beetle_D_N_A_Lab) and using primer pairs C1-J-2183 (5’-CAA CAT TTA TTT TGA TTT TTT GG-3’; *Jerry*) and TL2-N-3014 (5’-TCC AAT GCA CTA ATC TGC CAT ATT A-3’; *Pat*) (Simon et al. 1994) and *Mango Taq* DNA polymerase (Bioline, Luckenwalde, Germany). The PCR temperature progression was set: 30 s at 94 °C, 30 s at 47 °C, 60 s at 72 °C (× 35 cycles), 600 s at 72 °C. Amplification products were purified with Qiagen Qiaquick PCR purification columns (Qiagen, Hilden, Germany). Cycle sequencing was performed as follows: 15 s at 96 °C, 15 s at 50 °C, and 240 s at 60 °C (x 35 cycles) using PCR primers with BigDye Terminator v3.1 Cycle Sequencing Kit (Applied Biosystems, Foster City, California, USA). The sequencing products were purified by ethanol precipitation (25 µl of cold (-20°C) 99% ethanol, 2.5 µl of 3M sodium acetate added to product; centrifuged; washed with 25 µl of 70% ethanol), and additionally with Agencourt CleanSEQ (Agencourt Bioscience, Beverly, Massachusetts, USA) following protocol 000600v32 (Agencourt Bioscience 2006) before electrophoresis.

The DNA extraction of three specimens was additionally used for the amplification a central part of the cytochrome b apoenzyme (*cob*) gene by using the primer pair 5’-GAG GAG CAA CTG TAA TTA CTA A-3’ (CB3) and 5’-AAA AGA AA(AG) TAT CAT TCA GGT TGA AT-3’ (CB4) (Baraclough et al. 1999). This was done to prove assignment of adult and larval stages of one species for which *cox1* data were insufficient.

### Phylogenetic analysis

Additional *cox1* sequences of Philippine *Ancyronyx* species (Freitag and Balke 2011) previously submitted to ENA/GenBank (http://www.ebi.ac.uk/ena/) were included (see Table 1). The same applies for *Podelmis viridiaenea* Jäch, 1982 (Elmidae) from Sri Lanka that was used as outgroup. The newly amplified sequences were traced and aligned in CLUSTALW (Thompson et al. 1994) using BIOEDIT version 7.0.5.2. (Hall 1999) and default parameters. Phylogenetic analyses were conducted with MRBAYES vers. 3.1.2 (Ronquist et al. 2012) using the GTR (General Time Reversible) model (Tavaré 1986) with default priors starting with random trees with three heated and one cold Markov chains. The analysis was run by 1,000,000 generations, and the first 25% of samples from the cold chain have been discarded as burnin. Branch support for the Bayesian trees was assessed with posterior probabilities determined via the 50% majority rule consensus. This easy analysis is only intended for matching larva and adults of the species treated in this paper.

**Table 1.** ENA/GenBank accession numbers of DNA sequences, geographical origins, collection sites and organismic sample references of specimens used for molecular-genetic analyses.

**Species**	**Stage**	**Locality**	**Site**	**Voucher**	**cox1**	**cob**
*Ancyronyx jaechi* Freitag, 2012	adult	Sri Lanka	1	ZSM FR 027	HF937369	-
*Ancyronyx schillhammeri* Jäch, 1994	adult	Mindoro	303a	ZSM FR 029	HF937371	-
*Ancyronyx schillhammeri* Jäch, 1994	larva	Mindoro	303a	ZSM FR 030	HF937370	-
*Ancyronyx tamaraw* Freitag	adult	Mindoro	302	ZSM FR 011	HF937374	-
*Ancyronyx tamaraw* Freitag	larva	Mindoro	302	ZSM FR 012	HF937373	-
*Ancyronyx buhid* Freitag	adult	Mindoro	318	ZSM FR 088	-	HF937366
*Ancyronyx buhid* Freitag	larva	Mindoro	HR2g	ZSM FR 090	HF937375	HF937367
*Ancyronyx buhid* Freitag	larva	Mindoro	310a	ZSM FR 091	HF937376	HF937368
*Ancyronyx minerva* Freitag & Jäch, 2007	adult	Mindoro	303a	ZSM FR 033	HF937372	-
*Ancyronyx minerva* Freitag & Jäch, 2007	larva	Palawan	159	ZSM FR 025	HE588180	-
*Ancyronyx helgeschneideri* Freitag & Jäch, 2007	adult	Palawan	CR4	ZSM FR 007	HE588167	HE588183
*Ancyronyx procerus* Jäch, 1994	larva	Busuanga	169	ZSM FR 014	HE588171	HE588182
*Ancyronyx montanus* Freitag & Balke, 2011	larva	Palawan	16h	ZSM FR 038	HE588175	-
*Ancyronyx punkti* Freitag & Jäch, 2007	adult	Palawan	154	ZSM FR 008	HE588169	-
*Ancyronyx pseudopatrolus* Freitag & Jäch, 2007	adult	Palawan	16f	ZSM FR 003	HE588172	-
*Ancyronyx patrolus* Freitag & Jäch, 2007	adult	Busuanga	165	ZSM FR 032	HE588178	-
*Podelmis viridiaenea* Jäch, 1982	adult	Sri Lanka	1	ZSM FR 035	HE588181	-

### Morphological analysis

Digital photographs were taken with an OLYMPUS SZ 61 stereo microscope (species habitus), and an OLYMPUS CX 21 compound microscope (dissected body parts), both with digital photo adapter LW Scientific MiniVID DCM 310. For each illustration a series of photographs taken at various focus layers was stacked using the stack function (species habitus) and corrected weighted average function (dissected body parts) of COMBINEZM software (Hadley 2008). The same optical systems we used for the dissection of adult specimens and the material examination. Biometric measurements were done by the use of a calibrated ocular micrometer.

Scanning electron microscope (SEM) images of vacuum dried material were obtained using a ZEISS EVO 50 XVP. Except for the single larval specimen of *Ancyronyx schillhammeri*, all specimens were coated with gold using one dissected and one entire specimen each.

For all larval material examined, measurements of the head capsule width are given in mm (e.g. 1 L (0.31)) as a suitable indicator for the larval size and the instar stage assignment (see Freitag and Balke 2011).

Morphological terminology follows Kodada and Jäch (2005) and Freitag and Balke (2011).

### Abbreviations and repositories

Brgy.Barangay (local government unit district)

CLcalculated length (PL + EL)

ELelytral length

EWelytral width

HWhead width

IDinterocular distance

Llarva / larvae

Mmanual collection

MWmaximum pronotal width

Oc.Occidental

Or.Oriental

PHILPhilippines

PLpronotal length

sec.veget.surrounded by secondary vegetation

subm.submerged

CFMCollection Hendrik Freitag, Manila, Philippines, currently deposited at Ateneo de Manila University, Philippines

CZWCollection Herbert & Salvacion V. Zettel, Vienna, Austria

NMWNatural History Museum Vienna, Austria

PNMPhilippine National Museum Manila, Philippines

SMTDSenckenberg Museum of Zoology Dresden, Germany

ZMUCZoological Museum of the University Copenhagen, Denmark

ZSMZoological State Collections Munich, Germany

## Data resources

The data underpinning the analysis reported in this paper are deposited at GBIF, the Global Biodiversity Information Facility, http://ipt.pensoft.net/ipt/resource.do?r=ancyronyx_mindoro_data

All DNA sequences were submitted to ENA/GenBank via online submission to EMBL-EBI. Accession numbers and curatory information are listed in Table 1.

## Results

### DNA sequence analysis

Alignment of the *cox1* data and trimming ambiguous bases at the 3’ and 5’ ends yielded a matrix of 804 bp. None of the sequences contained indels. The sequences of the larvae of *Ancyronyx buhid* had nine ambiguous positions in-between which were coded as ‘N’s.

All adults and larvae could be matched unambiguously. Sequences of adult and larva of the same species from the same locality or island were identical or varied just in one base pair. Sequence samples of *Ancyronyx minerva* from Mindoro diverged in eight base pair positions (six of them synonymous substitutions) from that of the same species from Palawan.

The sequencing of *cox1* of the adults of *Ancyronyx buhid* failed and is not included in the phylogenetic analysis. The *cob* sequences, however, which were amplified for two larvae and an adult of this species allowed unambiguous matching of the developmental stages. Their aligned and trimmed particial *cob* sequences of 350 bp were identical except for four positions where a synonymous substitution was seen in one of either sequences.

**Figure 1.**
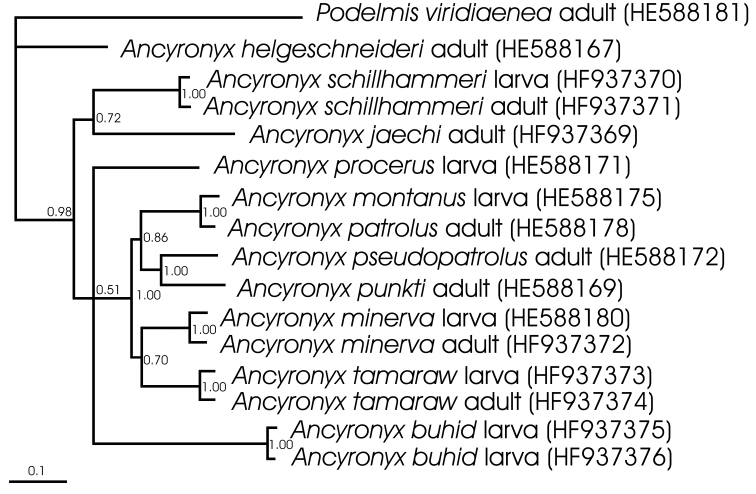
Phylogram of the consensus tree of the Bayesian analysis with branch lengths measured in expected substitutions per site. Posterior probability values (printed when > 0.5) at respective branches. Sample labels with developmental stage and ENA/GenBank code.

A 50% majority rule consensus trees based on *cox1* data is illustrated in Fig. 1. All samples of the same species clustered together, supported by 1.0 posterior probability values. The species of the *Ancyronyx patrolus* species group and its two subgroups respectively clustered together, however partly with lower posterior probability value support. *Ancyronyx buhid* does not cluster with the *Ancyronyx patrolus* group.

## Taxonomy

### Ancyronyx minerva

Freitag & Jäch, 2007

http://species-id.net/wiki/Ancyronyx_minerva\according to Freitag 2013

Figs 2, 5

Ancyronyx minerva Freitag & Jäch, 2007: 50–53 (adult description); Freitag and Pangantihon 2010: 133 (first record Mindoro); Freitag and Balke 2011: 53–58 (larva description), 79 (key); Freitag 2012: 63 (world check list).

#### Material examined.

1 ♀, 6 L (0.22; 0.25, 2 × 0.29, 0.31) (CFM) “PHIL.: Mindoro, Puerto Galera, NR km 37.2, downstr. Tamaraw Falls; riffle& fall; rocks, woodlitter, roots; sec.veget.; c. 80m asl., 13°27'03"N, 120°59'27"E 22.4.1994, leg. Freitag (302)M”; 1 ♀ [FR015], 1 ♂ [FR033], 1 L (0.31) (ZSM) “PHIL.: Mindoro, San Teodoro, Tukuran Riv.; small lowld.riv.; riffle & run; woodlitter, gravel; sec.veget.; c. 30m asl., 13°25'34"N, 120°58'37"E 23.4.1994, leg. Freitag (303a)M”; 1 ♂ (CFM) “PHIL.: Mindoro, Puerto Galera, NR km 59, downstr. Aninuan Falls; riffle; small mount. riv., boulder, rocks, gravel, woodlitter; sec.veget.; c. 80m asl., 13°29'10"N, 120°54'18"E 24.4.2009, leg. Freitag (304)M”; 1 ♀ pter., 2 L (0.21, 0.25 [FR079, FR080]) (ZSM) “PHIL.: Mindoro Oriental, Municipality Victoria, Brgy. Malayas, Malayas River; W Naujan tributary; sec. veget., submerged wood, riffle, c. 20 m asl., c. 13°09'26"N, 121°18'29"E; 22.2.2010 leg. Freitag & Pangantihon (308a)M”; 1 ♂ pter. (CFM) “PHIL.: Mindoro Oriental, Bongabong, Brgy. Formon, Pastuhan, Tangisan Falls; deep mountain valley, sec. forest, submerged wood, riffle, c. 200 m asl., c. 12°43'N, 121°23'E; 27.10.2011 leg. Freitag (318a)M”; 1 ♀, 2 L (0.24 [FR087], 0.27) (ZSM, CFM) “PHIL.: Mindoro Oriental, Bongabong, Brgy. Formon, Pastuhan, Tangisan Falls; deep mountain valley, sec. forest, gravel & boulders, riffle, c. 200 m asl., c. 12°43'N, 121°23'E; 27.10.2011 leg. Freitag (318)M”; 1 ♀ (CFM) “PHIL: Or. Mindoro, Roxas, Brgy. San Vicente, Baroc River; subm. wood; gravel flood plains; c. 12°37'07"N, 121°24'06"E, 90m asl; leg. Freitag 1 Apr.2013 (BRf)M”; 1 ♂, 1 ♀ (CFM) “PHIL: Or. Mindoro, Roxas, Brgy. San Vicente proper, Taugad River; subm. wood; sec.veget.; c. 12°37'06"N, 121°23'49"E, 100m asl; leg. Freitag 2 Apr.2013 (TR1f)M”; 1 ♂, 1 ♀ 3 L (0.29, 2 × 0.31) (CFM) “PHIL: Or.Mindoro, Roxas, Brgy. San Vicente, Tauga River; rocks, riffle & run; sec.veget.; c. 12°37'18"N, 121°22'58"E, c. 140m asl; leg. Freitag 17.11.2011 (TR2g)M”; 1♂, 1♀, 3 L (0.27, 0.29, 0.31) (CFM): same locality and microhabitat “leg. Freitag & Pangantihon 07.7.2012 (TR2g)M”; 1 ♀ (CFM) “PHIL: Or. Mindoro, Roxas, Brgy. San Vicente, Baroc River tributary Hiyong Creek; bottom gravel, run & riffles; sec.veget.; c. 12°37'27"N, 121°22'48"E, 147m asl; leg. Pangantihon, 29.Jun.2012 (THCc)M”; 2 L (0.24, 0.25) (CFM) “PHIL: Or. Mindoro, Roxas, Brgy. San Vicente, Baroc River tributary Hiyong Creek; subm. root packs, run; sec.veget.; c. 12°37'27"N, 121°22'48"E, 147m asl; leg. Freitag & Pangantihon, 07.Jul.2012 (THCh)M”; 1 L (0.31) (CFM) “PHIL: Or. Mindoro, Roxas, Brgy. San Vicente, Sitio Quirao, Hinundugan tributary Quirao Buhay Creek; rocks, run; 12°36'10"N, 121°23'00"E, 130m asl; leg. Freitag & Pangantihon, 30.06.2012 (HBCg)M”.

#### Distribution.

The species is known from Busuanga, Mindoro and Palawan (Philippines) and is common on these islands.

**Figures 2–9.**
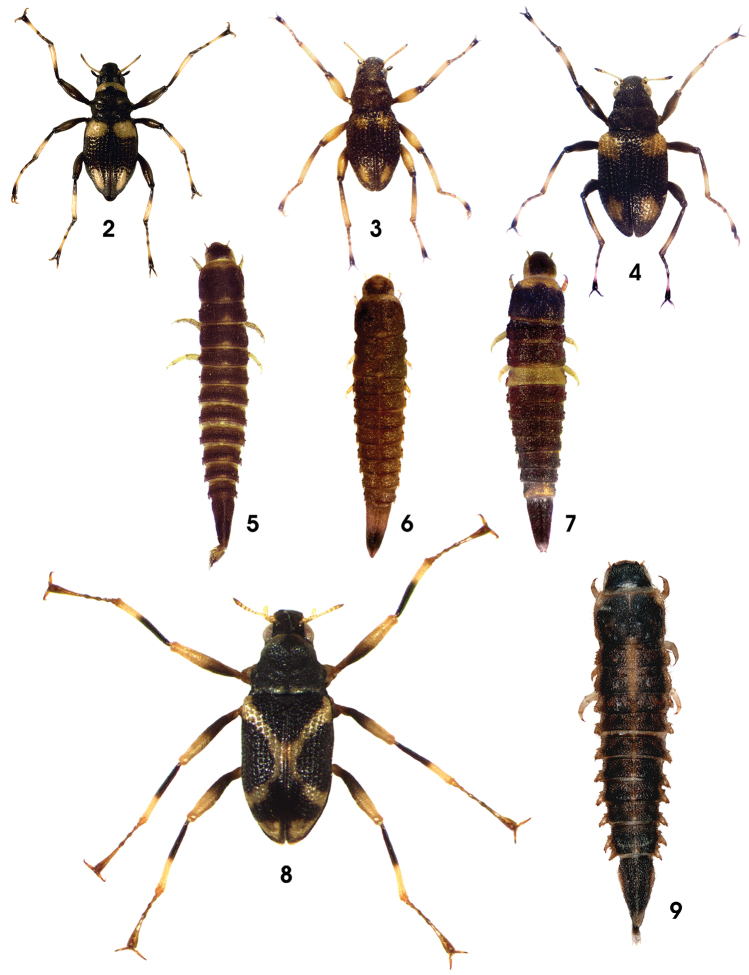
Habitus (not to scale) of **2** *Ancyronyx minerva*, adult **3** *Ancyronyx tamaraw*, sp. n. adult **4** *Ancyronyx buhid*, sp. n., adult **5** *Ancyronyx minerva*, larva **6** *Ancyronyx tamaraw*, sp. n., larva **7** *Ancyronyx buhid*, sp. n., larva **8** *Ancyronyx schillhammeri*, adult **9** *Ancyronyx schillhammeri*, larva.

#### Remarks.

Morphological variations between the population of different islands are not evident, but the *cox1* gene varies in a few more substitutional sites than within a population of one and the same island. The larva was described and illustrated in Freitag and Balke (2011).

#### Ecology.

Both, adults and larvae, are usually collected from boulder and rock surfaces, or submerged rootpacks in run and riffle sections. The species is predominantly found in clean, small to medium sized permanent streams. (Freitag and Pangantihon 2010, Freitag and Jäch 2007).

### Ancyronyx tamaraw

Freitag sp. n.

http://zoobank.org/9D03899B-56E5-4E7C-9C0E-A5154E30B2FC

http://species-id.net/wiki/Ancyronyx_tamaraw

**Figure 10.**
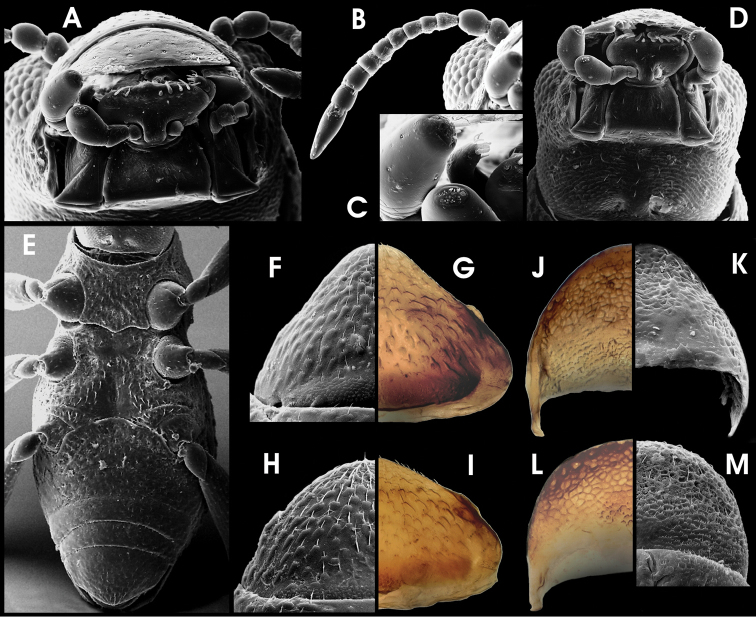
*Ancyronyx tamaraw* Freitag, sp. n., (SEM photographs in greyscale, stereo microscope photographs in colour; not to scale: see respective chapter for size measurements); adult male: **A** head, frontal **B** antenna, frontal **C** maxillary and labial palpi, frontal **D** head, ventral **E** throax and abdomen, ventral; adult female: **F, G** ventrite 5, ventral; adult male: **H, I** ventrite 5, ventral; adult female: **J, K** tergite VIII, dorsal; adult male: **L, M** tergite VIII, dorsal.

**Figure 11.**
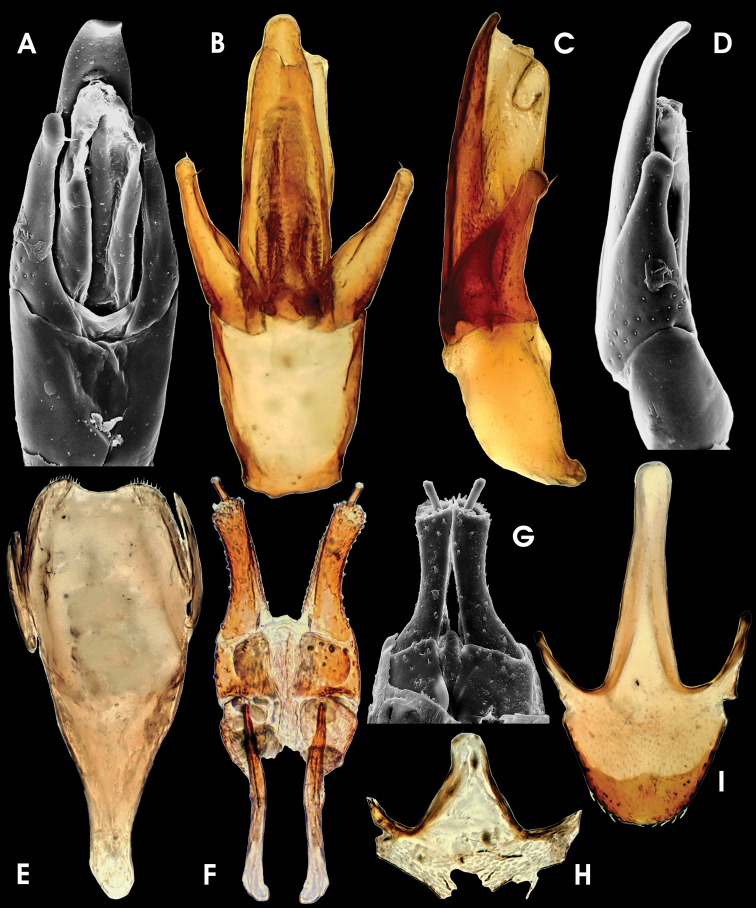
*Ancyronyx tamaraw* Freitag, sp. n., (SEM photographs in greyscale, stereo microscope photographs in colour; not to scale: see respective chapter for size measurements); adult male: **A** aedeagus, ventral **B** aedeagus, dorsal **C, D** aedeagus, lateral **E** sternite IX, ventral; adult female: **F, G** ovipositor, ventral; adult male: **H** sternite VIII, ventral; adult female: **I** sternite VIII, ventral.

**Figure 12.**
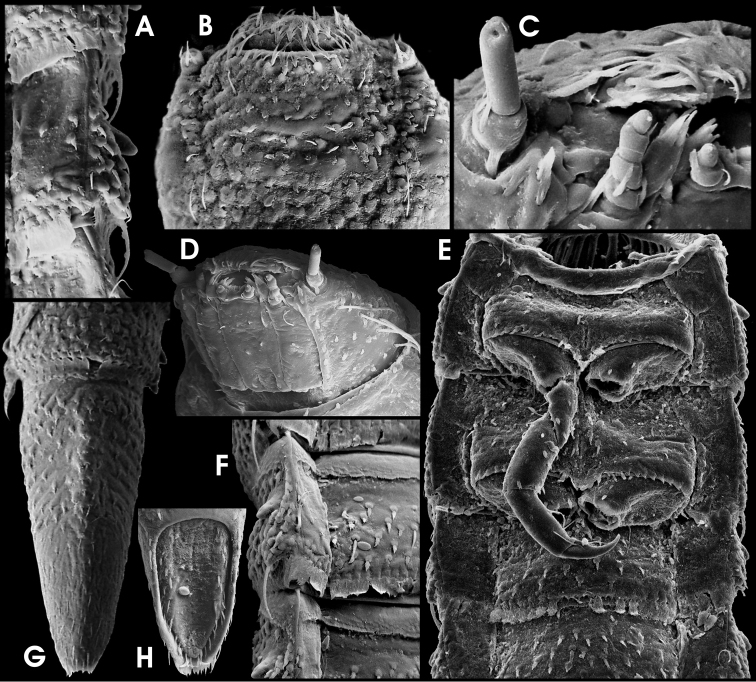
*Ancyronyx tamaraw* Freitag, sp. n., larva (SEM photographs; not to scale): **A** detail of abdominal segment III, lateral, with posterolateral projections and spiracles **B** head, dorsal **C** head, frontal **D** head, ventrolateral **E** meso- and metathoracic and first and second abdominal segments, ventral **F** lateral parts of median sclerites of abdominal venter, with setiferous tubercles and lateral projections **G** abdominal segments VIII and IX, dorsal **H** operculum, ventral.

Figs 3, 6 , 10A–M , 11A–I , 12A–H

#### Etymology.

This small and probably rare species is named in reference to its type locality, the Tamaraw Falls on the island of Mindoro. The tamaraw is a small Mindoro-endemic buffalo. The term is used as noun in apposition.

#### Type material.

**Holotype**♂ (NMW) “PHIL: Mindoro, Puerto Galera, NR km 37.2 downstr. Tamaraw Falls; riffle & fall; rocks, woodlitter, roots; sec.veget.; c. 80m asl., 13°27'03"N, 120°59'27"E 22.4.1994, leg. Freitag (302)M”, terminal parts of abdomen incl. aedeagus glued separately. **Paratypes:** 18 ♀♀, 15 ♂♂ (NMW, ZSM [FR011], ZMUC, SMTD, CFM), 9 L (0.24, 0.28, 5 × 0.29, 2 × 0.30) (ZSM [FR012], NMW, SMTD, CFM): same label data as holotype. 3 ♀♀, 4 ♂♂ (PNM): same locality data as holotype, leg. Freitag 11.2.2013.

#### Adult description.

Body 1.2–1.5 mm long (CL + exposed portions of head & tergit VIII); CL: 1.02–1.36 mm; EW: 0.51–0.58 mm, CL/EW: 2.0–2.4.

Colouration as in Fig. 3: ventral side, coxae, trochanter, and pronotum brown; entire dorsal head capsule and mouthparts dark brown; elytra dark brown except for two pairs of yellow patches; anterior yellow elytral patches round, extending each between first and third row of elytral punctures, not reaching median or anterior elytral margin; posterior yellow elytral patches elongate-oval to subtriangular, not reaching median, lateral, and apical elytral margin; antennae yellow (except for dark tips and scape); legs dominantly yellowish except for coxa, trochanter and brown areas around all articulations, especially proximal and distal areas of femur, proximal third of tibia and distal third of fifth tarsomere.

HW 0.29–0.35 mm; ID 0.15–0.18 mm; labrum and distal portion of clypeus moderately densely micropunctate and covered with short trichoid setae (Fig. 10A); proximal portion of clypeus and frons microreticulate and punctate; frontoclypeal suture inconspicuous, slightly convex. Eyes slightly protruding. Antennae (Fig. 10B) with 11 antennomeres, slender, c. as long as head wide. Genae (Fig. 10D) rugose and reticulate, with indistinct pubescence. Gula (Fig. 10D) with somewhat regularly arranged striae except for central portion, moderately densely pubscent; gular sutures absent. Mandible with bilobed tip. Maxilla (Fig. 10A) with very short cardo; stipes with distinct flat, triangular elevation ventrally; galea palp-like elongate, with apical setae and sensilla (Fig. 10C); lacinia not examined. Labium (Fig. 10A) with subtrapezoidal postmentum, prementum suboval, undivided, with subapical row of ten short trichoid setae; labial palps three-segmented, c. as long as postmentum, with apical setae and sensilla (Fig. 10C).

Pronotum (Fig. 3) 0.35–0.38 mm long (PL), 0.38–0.41 mm wide (MW), inconspicuously wider than long (PL/MW), widest at about posterior 0.4, distinctly narrower than elytra, with moderately deep transverse groove; anteriorly of transverse groove slightly vaulted; posterior portion medially elevated; posterolateral oblique grooves small and round, but conspicuous; lateral margin distinctly arcuate; anterior margin convex; pronotal surface entirely microreticulate and with moderately densely arranged seta-bearing tubercles; lateral pronotal carina absent; hypomeron inconspicuously reticulate. Prosternum (Fig. 10E) punctate; prosternal process broadly subpentagonal, distinctly wider than long, almost flat.

Metascutellum subcordiform, medially slightly impressed, micropunctate. Elytra (Fig. 3) elongate, 0.78–0.98 mm long (EL), c. 1.5–1.7 times as long as wide (EL/EW), laterally arcuate (broadest at about anterior 0.45), anteriorly slightly roundly convergent, posteriorly roundly convergent to apices, with eight longitudinal, moderately impressed rows of punctures (counted at level of metacoxae); median rows more regular than lateral ones; five strial rows between suture and humerus; punctures large and moderately deeply impressed; interstices and intervals convex, granulose to microreticulate; lateral elytral gutter very narrow, inconspicuous; humeri broadly rounded; elytral apices separately rounded. Mesoventrite (Fig. 10E) short, micropunctuate, with a round median impression and a sublateral pair of round elevations. Metaventrite (Fig. 10E) comparably small; medial impression wide, not conspicuously longitudinal, rather a shallow, funnel-like round impression deepest at median posterior margin; disc with scattered inconspicuous setose tubercles, glabrous in-between. Anepisternum 3 microreticulate with additional scattered punctures. No hind wings present in all specimens examined.

Legs (Fig. 3) slightly longer than body; coxae large; pro- and mesocoxae (Fig. 10E) subglobular (drop-shaped), lateral portion visible in dorsal view; metacoxae (Fig. 10E) rather conically elevated from a flat base, not visible in dorsal view; trochanter (Fig. 10E) small, broadly lanceolate, invisible in dorsal view; femora and tibiae with micro-setiferous tubercles; tibiae distally with few trichoid setae; each tarsomere with ventral pair of short trichoid setae; claws moderately wide, rather short (compared to other *Ancyronyx* species), strongly bent, base of each with two teeth, distal one distinctly larger.

Ventrite 1 (Fig. 10E) distinctly arcuately projected anteriad; medioanterior portion depressed (connecting to funnel-like metaventrite impression). Ventrites 2–4 (Fig. 10E) with small, moderately densely arranged punctures; surface between punctures glabrous; tubercles larger and denser toward lateral declivity; ventrite 5 (Figs 10F–I) moderately densely covered with short adpressed setae emerging from flat tubercles; lateral projection inconspicuous.

Aedeagus (Figs 11A–D) 410 µm long, somewhat similar to that of *Ancyronyx sophiemarie* Jäch, 2004 (see Jäch 2004: figs 3–4), but phallobase longer and apical area of median lobe distinctly wider. Phallobase almost symmetrical, more or less straight, except for tapered and ventrally bent base, slightly longer ventrally, with conspicuous, strongly sclerotised ventral and lateral margins. Median lobe moderately long and wide (c. 70 µm), straight, evenly and slightly tapering towards apex up to apical 0.2 of median lobe, then moderately bent ventrad and more abruptly tapering into a wide apical area; apex pointing ventrad, with numerous pore-like structures on dorsal side; basolateral (penile) apophyses short, not overreaching paramere base; ventral sac distinct, ventrally protruding (Fig. 11C), internal portion speckled, moderately sclerotised (Fig. 11B). Fibula weakly sclerotised; corona inconspicuous. Parameres short, c. 145 µm long, reaching about basal 0.7 of aedeagus, elongately subtriangular, widely separated ventrally; laterobasal margin emarginate (Fig. 11D) apical portion roundly widened, narrowest subapically, with few short setae (two apical, two medio-subapical).

Sternite IX (Fig. 11E), c. 380 µm long, with short anterior strut, not clearly partitioned from apical portion; apical corners rounded, with patches of micro-setae, apical margin broadly emarginate; longer paraproct reaching apical margin.

Ovipositor (Fig. 11F, G) c. 480 µm long. Stylus slender, rather staight, with various sensilla. Coxite long, outer margin curved, distal portion with several rather short and broad, peg-like spines, most densely set subapically at lateral margins; inner margin pubescent; basal portion with similar, slightly slenderer, peg-like spines; near valvifer insertation with dense patch of very small spines. Valvifer as long as coxite; fibula distinctly bent and widened caudally.

Secondary sexual characters: Sternite VIII in male (Fig. 11H) short, weakly sclerotised and with very short median strut; in female (Fig. 11I) distinctly longer, slightly more sclerotised than in male, apical corners broadly rounded and with small seate, median portion with dense micro-pubescence (not conspicuous in males). Tergite VIII in female (Figs 10J, K) long, subtriangular, slightly longer than broad (c. 220 µm long, 210 µm wide), with few moderately short setae (apical ones widest), condyles large and conspicuous. Tergite VIII in male (Figs 10L, M) subsemicircular, distinctly wider than long and shorter than in female (c. 180 µm long, 225 µm wide); in apical half with moderately long setae. Ventrite 5 in female (Figs 10F, G) subtriangular (c. 250 µm long, 360 µm wide); in male (Figs 10H, I) similar in general shape, but slightly shorter (c. 210 µm long, 370 µm wide) and rounder.

#### Adult differential diagnosis.

*Ancyronyx tamaraw* superficially resembles *Ancyronyx sophiemarie* from Sibuyan and *Ancyronyx minerva*. The new species can be easily distinguished by the combination of elytral colour pattern (anterior yellow elytral patches circularly round, not reaching median or anterior elytral margin; posterior patches elongate-oval to subtriangular), the predominantly yellowish legs, the brown (not black) pronotum and head, and it’s aedeagus with wide and flat apical portion.

#### Larval diagnosis

**(based on sixth instar).** Colour (Fig. 6) similar to that of *Ancyronyx minerva* (see Freitag and Balke 2011: figs 3, 11A–L), but most distinctly different by anterior median head portions (clypeus, anterior frons) pale; anterior yellow pronotal band small, limited to very most anterior portion; at least pro-, meso-, metanotum with small circular-round (not broadly subtriangular) yellow pattern at medioposterior margin; abdominal segment IX with pale yellowish apex and a conspicuous dark pattern extending c. posterior 0.2–0.4; abdominal segment IX relatively longer than in larvae of *Ancyronyx minerva*.

HW 0.29 mm; entire larva about 2.7 mm long. Body elongate very similar in the external characters to that of *Ancyronyx minerva*, except for the following: Posterolateral projections (Figs 12A, F) of all abdominal segments short, generally not overreaching posterior segment margins.

Head (Figs 6, 12B–D) with subparallel lateral margins posterior 0.1–0.7, moderately tapering anteriad; lateral setae long; a dorsolateral pair of moderately long single setae present (Fig. 12B). Frontal suture distinctly V-shaped. Labrum subtrapezoidal. Antennae (Figs 12C, D) c. ¼ as long as head; peduncle at with at least one faciculate seta; pedicel c. two times as long as scape, c. three times as long as wide. Maxillary stipes (Fig. 12D) slightly tapering towards apex. Labial mentum (Fig. 12D) narrowest basal; lateroapical pair of spines rather small, positioned at distal edge.

Pro-, meso-, metathorax and legs (Figs 6, 12E) almost as in *A minerva*. Pronotum with rather inconspicuous small round signa (glabrous areas) in posterior half. Ventral sclerites of thorax (Fig. 12E) rugulose, not glabrous.

Abdomen (Figs 6, 12A, E–H) without conspicuous dorsosagittal carinae except for the anterior half of segment IX (Fig. 12G); squamose setae at posterior rim of segments I–VIII large (Figs 12E, F). Ventral sclerites of segment I with distinct sagittal ridge in anterior half, reaching c. ½ to ⅔ of segment length. Apex of segment IX (Fig. 12G) emarginate (sometimes inconspicuous due to apical setae). Operculum (Fig. 12H) longer than in *Ancyronyx minerva* (more than double as long as wide).

#### Larval differential diagnosis.

The species can most easily be distinguished from *Ancyronyx minerva* which looks superficially most similar by the partly pale colour pattern of the dorsal head, the narrower circular medioposterior pale pattern at pro-, meso-, and metanotum and the longer last abdominal segment with pale apical area and distinctly dark subapical portion.

#### Variation between larval instars.

The final and prefinal instar stages available for this study do not vary conspicuously except for their size.

#### Distribution.

The species is known from the type locality in north-eastern Oriental Mindoro. Additional material that appears conspecific is known from Subic, Zambales, Luzon and Bohol (unpublished material at CFM and NMW).

#### Ecology.

The specimens were collected in well oxygenated water from rock surfaces, submerged woodlitter and roots in run and riffle sections of the stream below Tamaraw Falls. Since all examined material comes from this, in fact clean and almost natural small mountain river, any detailed conclusion about the habitat and ecological requirements would be highly speculative. It is, however, surprising that not any single specimen was found at any other collection site in Mindoro so far.

### Ancyronyx buhid

Freitag sp. n.

http://zoobank.org/C731192C-EF06-4BDD-A387-9AB967DC9FED

http://species-id.net/wiki/Ancyronyx_buhid

**Figure 13.**
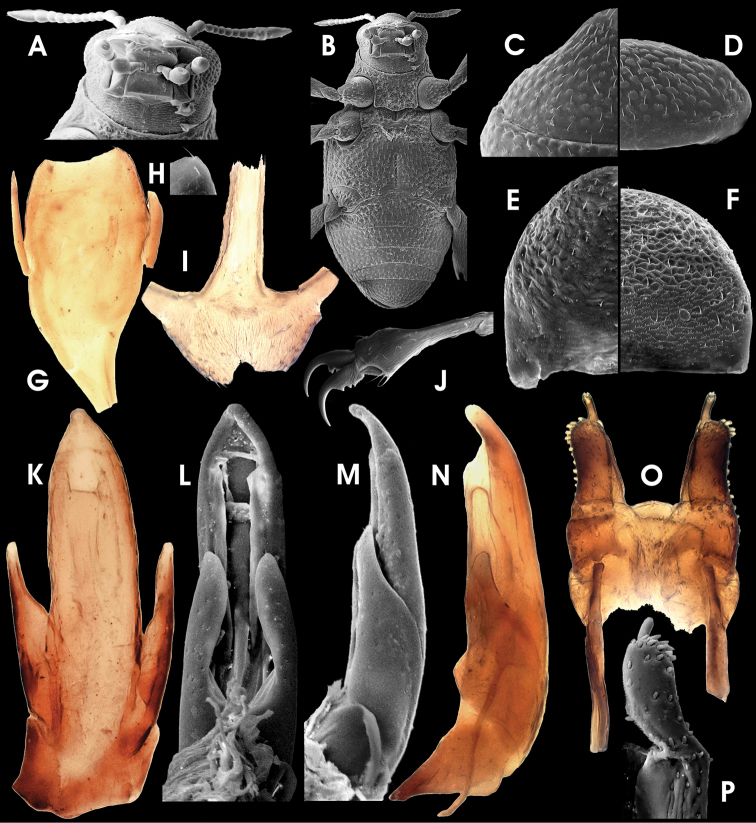
*Ancyronyx buhid* Freitag, sp. n., (SEM photographs in greyscale, stereo microscope photographs in colour; not to scale); adult male: **A** head, ventral **B** entire body, ventral **C** adult female: ventrite 5, ventral; adult male: **D** ventrite 5, ventral; adult female: **E** tergite VIII, dorsal; adult male: **F** tergite VIII, dorsal **G** sternite IX, ventral; aedeagus, ventral; adult female: **I** sternite VIII, ventral; adult male: **J** proximal tarsal segment, lateral **K** aedeagus, dorsal **L** aedeagus, ventral; **M, N** aedeagus, lateral **E** adult female: **O, P** ovipositor, ventral.

**Figure 14.**
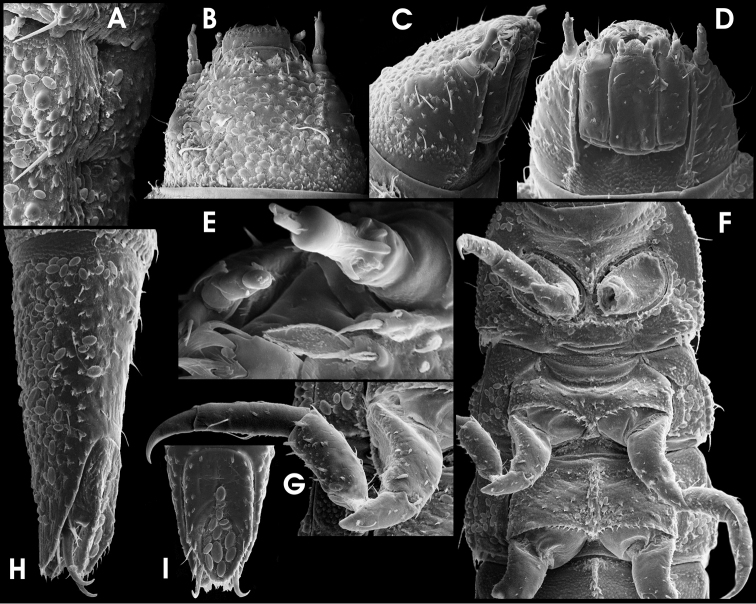
*Ancyronyx buhid* Freitag, sp. n., larva (SEM photographs; not to scale): **A** detail of abdominal segment VII, lateral, with posterolateral projections and spiracles **B** head, dorsal **C** head, lateral **D** head, ventral **E** antenna, frontal **F** thoracic and first abdominal segments, ventral **G** midleg, ventral **H** abdominal segment IX, lateral **I** operculum, ventral.

Figs 4, 7 , 13A–P , 14A–I

#### Etymology.

The species is named for the indigenous ethnic group of the Buhid in whose ancestral areas it commonly occurs. Same time, their kind support and care during regular field trips of faculty members and students of the Ateneo de Manila University’s Biology Department to the their Ancestral Domain should be honoured. Buhid is used as noun in apposition.

#### Type material.

**Holotype**♂ (NMW) “leg. Jäch 1.12. \ PHILIPPINEN – Mindoro \ 20km W Calapan 1992 \ Hidden Paradise (21)”, terminal parts of abdomen incl. aedeagus glued separately. **Paratypes**: 11 ♂♂, 23 ♀♀, 6 L (3 × 0.29, 2 × 0.30, 0.31) (NMW): same data as holotype. 8 L (2 × 0.29, 0.30, 4 × 0.31, 0.32) (NMW) “PHILIPPINEN – Mindoro \ 20km W Calapan 1992 \ Hidden Parad. 20.–21.11. \ leg. Jäch(10)”; 4 ♀♀ “PHILIPPINEN – Mindoro \ 20km W Calapan 1992 \ Hidden Parad. 20.–21.11. \ leg. Schillhammer (10)”; 3 ♂♂, 3 ♀♀ (CZW) “PHIL.: Mindoro or. \ Baco, Hidden Parad. \ 19.–20.11.1993 \ leg. Zettel (27)”; 7 ♂♂, 4 ♀♀, 4 L (0.27[FR091]), 2 × 0.31, 0.29) (SMTD) “PHIL.: Mindoro, Baco, Dulangan, Lantuyan torrent mount.Riv.; sec.veget.; riffle, wood debris, c. 55m asl., c. 13°16'08"N, 121°04'56"E 02.4.2000, leg. Freitag (310a)M”; 1 ♂, 3 ♀♀ (PNM, ZSM [FR088]) “PHIL.: Mindoro Oriental, Bongabong, Brgy. Formon, Pastuhan, Tangisan Falls; deep mountain valley, sec. forest, submerged wood, riffle, c. 200 m asl., c. 12°43'N, 121°23'E; 27.10.2011 leg. Freitag (318)M”; 11 L (0.22, 0.24, 0.25, 6 × 0.31, 2 × 0.32) (CFM) “PHIL: Or. Mindoro, Roxas, Brgy. San Vicente, Tauga River; rocks, riffle & run; sec.veget.; c. 12°37'18"N, 121°22'58"E, c. 140m asl; leg. Freitag 17.3.2012 (TR2g)M”; 1 ♂ [FR086] (ZSM), 4 L (0.21, 0.24, 0.29, 0.31) (CFM) “PHIL: Or. Mindoro, Roxas, Brgy. San Vicente, Tauga River; rocks, riffle & run; sec.veget.; c. 12°37'18"N, 121°22'58"E, c. 140m asl; leg. Freitag & Pangantihon 07.7.2012 (TR2g)M”; 3 ♂♂, 8 ♀♀ (SMTD) “PHIL: Or. Mindoro, Roxas, Brgy. San Vicente, Tauga River; subm. wood, run; sec.veget.; c. 12°37'18"N, 121°22'58"E, c. 140m asl; leg. Pangantihon, 22 Jan.2013 (TR2f)M”; 2 ♂♂, 2 ♀♀ (ZSM) “PHIL: Or. Mindoro, Roxas, Brgy. San Vicente, Sitio Tauga Diit, Baroc River tributary Tauga Diit; subm. wood, run & riffle; sec.veget.; 12°37'32"N, 121°21'17"E, 180m asl; leg. Freitag & Pangantihon, 05.Feb.2012 (TIRf)M”; 2 ♀♀ (CFM) “PHIL: Or. Mindoro, Roxas, Brgy. San Vicente, Baroc River tributary Hiyong Creek; side pool, littoral sand and gravel; sec.veget.; c. 12°37'27"N, 121°22'48"E, 147m asl; leg. Freitag & Pangantihon, 05.Feb.2012 (THCe)M”; 6 L (0.22, 2 × 0.24, 0.27, 2 × 0.31) (NMW) “PHIL: Or. Mindoro, Roxas, Brgy. San Vicente, Baroc River tributary Hiyong Creek; subm. root packs, run; sec.veget.; c. 12°37'27"N, 121°22'48"E, 147m asl; leg. Freitag & Pangantihon, 07.Jul.2012 (THCh)M”; 3 ♂♂, 7 ♀♀, 3 L (0.27, 0.30, 0.31) (CFM) “PHIL: Or. Mindoro, Roxas, Brgy. San Vicente, Sitio Tauga Diit, Baroc River tributary Tauga Daka; subm. wood in run; sec.veget.; c. 12°37'56"N, 121°20'33"E, c. 350m asl; leg. Freitag, 4 Apr. 2013 (TDR2f)M”; 15 ♂♂, 14 ♀♀ (NMW) “PHIL: Or. Mindoro, Roxas, Brgy. San Vicente, Sitio Tauga Diit, Baroc River tributary Tauga Daka; subm. wood in run; sec.veget.; c. 12°38'05"N, 121°19'33"E, c. 530m asl; leg. Pangantihon, 18 Jan. 2013 (TDR3f)M”; 4 ♂♂, 7 ♀♀ (ZMUC) same locality and microhabitat “leg. Pangantihon, 23 Jan. 2013 (TDR3f)M”; 16 ♂♂, 14 ♀♀ (CFM) same locality and microhabitat “leg. Pangantihon, 15 Feb. 2013 (TDR3f)M”; 3 ♀♀, 2 L (0.27, 0.34) (PNM) “PHIL: Or. Mindoro, Roxas, Brgy. San Vicente, Sitio Tagaskan, Hinundugan River; rocks, run; sec.veget.; c. 12°35'22"N, 121°21'54"E, c. 200m asl; leg. Freitag & Pangantihon, 20.12.2011 (HR2g)M”; 4L (0.25 [FR090], 0.26, 2 × 0.31, 0.32) (ZSM): same locality and microhabitat “leg. Freitag & Pangantihon, 06.Feb.2012 (HR2g)M”; 2 ♂♂, 5 ♀♀ (CFM) “PHIL: Or. Mindoro, Roxas, Brgy. San Vicente, Sitio Tagaskan, Hinundungan River; subm. wood, run c. 12°36'30"N, 121°22'38"E, c. 200m asl; leg. Pangantihon; 31 Mar. 2013 (HR3f)M”; 6 ♂♂, 1 ♀, 9 L (2 × 0.24, 2 × 0.27, 3 × 0.30, 2 × 0.33) (CFM) “PHIL: Or. Mindoro, Roxas, Brgy. San Vicente, Sitio Tagaskan, Hinundungan River; rocks, riffle & run; c. 12°36'30"N, 121°22'38"E, c. 200m asl; leg. Freitag; 31 Mar. 2013 (HR3g)M”; 3 L (0.24, 0.25, 0.31) (CFM) “PHIL: Or. Mindoro, Roxas, Brgy. San Vicente, Sitio Quirao, Hinundugan tributary Quirao Buhay Creek; rocks, run; 12°36'10"N, 121°23'00"E, 130m asl; leg. Freitag & Pangantihon, 30.06.2012 (HBCg)M”; 1 L (0.30) (CFM) “PHIL: Or. Mindoro, Roxas, Brgy. San Vicente, Hinundugan tributary Tinggiwang Creek; subm. wood, run; 12°35'48"N, 121°22'00"E, c. 180m asl; leg. Freitag; 31 Mar.2013 (HTCf)M”. **Other material:** 1 ♂ (CFM) “PHIL: Or. Mindoro, Roxas, Brgy. San Vicente, Tauga River; subm. wood, run; sec.veget.; c. 12°37'18"N, 121°22'58"E, c. 140m asl; leg. Freitag 28.Nov.2011 (TR2f)M”.

#### Adult description.

Body 1.4–1.6 mm long (CL + exposed portions of head & tergit VIII); CL: 1.25–1.38 mm; CL/EW: 1.9–2.1. Colouration as in Fig. 4: entire dorsal head capsule, mouthparts, pronotum, and elytra (except for two pairs of yellow patches) black; anterior yellow elytral patches extending from humeri mediad approximately up to second row of elytral punctures, not reaching median elytral margin; posterior yellow elytral patches oval, not reaching median, lateral, and apical elytral margin; ventral side, coxae, trochanter, femur, proximal half of tibia, areas around tibial and tarsomere articulations, and claws brown; at least distal half of tibia and proximal portion of fifth tarsomere yellowish; antennae yellow (except for dark tips and basal segment).

Head (Figs 4, 13A) 0.33–0.36 mm wide (HW); ID 0.19–0.21 mm; labrum smooth, with moderately densely trichoid pubescence; clypeus (except for anterior margin) and frons with longitudinal striae on microreticulate ground, moderately densely covered with short trichoid setae; frontoclypeal suture straight and conspicuous. Eyes slightly protruding. Antennae (Fig. 13A) with 11 antennomeres, slender, slightly shorter than head width. Genae (Fig. 13A) reticulate, with indistinct pubescence. Gula (Fig. 13A) with regularly arranged, scale-like striae (including median portion), with inconspicuous pubscens; gular sutures absent. Mouthparts (Fig. 13A) almost as in *Ancyronyx tamaraw* except for shorter postmentum, that is rather sub-rectangular than trapezoidal; prementum with subapical row of eight very short trichoid setae; labial palps three-segmented, slightly longer then postmentum.

Pronotum (Fig. 7) 0.36–0.40 mm long (PL), 0.42–0.44 mm wide (MW), slightly wider than long (PL/MW), widest at about posterior 0.3, distinctly narrower than elytra, with deep transverse groove; anteriorly of transverse groove slightly vaulted; posterior portion broadly vaulted; posterolateral oblique grooves divided (two pairs), elongate, conspicuous; lateral margin distinctly arcuate; anterior margin distinctly convex; pronotal surface entirely microreticulate and rugose, with inconspicuous pubescence; lateral pronotal carina absent; hypomeron as pronotal surface. Prosternum (Fig. 13B) transverse, prosternal process broadly subpentagonal, distinctly wider than long, both appearing rugose by microreticulation superimposed with irregularly shaped setiferous tubercles.

Metascutellum subcordiform, micropunctate. Elytra (Fig. 4) broadly elongate, 0.89–0.98 mm long (EL), 0.61–0.66 mm wide (EW), c. 1.4–1.5 times as long as wide (EL/EW), almost parallel-sided in anterior 0.1–0.65, anteriorly slightly convergentposteriorly roundly convergent to apices, with c. nine longitudinal, moderately impressed rows of punctures (counted at level of metacoxae); median rows rather inconspicuous; lateral rows more regular and more deeply impressed than median rows; six to seven strial rows between suture and humerus; punctures moderately large and moderately deeply impressed, lateral punctures deeper than median ones; interstices and intervals granulose to micropunctate; lateral elytral gutter narrow; humeri roundly obtuse; elytral apices inconspicuously separately rounded.

Mesoventrite (Fig. 13B) very short, most anteriorly micropunctuate, posteriorly granulose, with deeply impressed median longitudinal impression. Metaventrite (Fig. 13B) large, without glabrous areas, entirely microreticulate superimposed with irregularly shaped setiferous tubercles; the latter appearing reticulately connected in lateral portions; tubercles smaller and shallower at disc; median longitudinal impression deeply impressed, laterally extending into a subtriangular groove; groove without setiferous tubercles. Anepisternum 3 microreticulate with one row of punctures. Hind wings present in all specimens examined, venation not examined.

Legs (Figs 4, 13B, J) approximately as long as body, or very little shorter; coxae large, only procoxae visible in dorsal view; pro- and mesocoxae (Fig. 13B) subglobular (drop-shaped); metacoxae (Fig. 13B) rather obtuse and shallowly elevated, obliquely conoidal; trochanter (Fig. 13B) short, broadly lanceolate, not visible in dorsal view, distal end distinctly pointed; femora and tibiae appearing longitudinally striated by dense cover with very elongate, micro-setiferous tubercles; tibiae distally with rather short and inconspicuous setae; tarsomeres with small scattered setae (Fig. 13J), most conspicuous at ventral side and near claw insertation; claws (Fig. 13J) large, rather slender, strongly bent; base of each with three teeth, distal one very large (mutilated in specimen figured in 13J).

Ventrite 1 (Fig. 13B) arcuately projected anteriad between hind coxae; microreticulate and tuberceliferous as in metaventrite especially near anterior margin. Ventrites 2 –4 (Fig. 13B) with evenly distributed, subcordiform, setiferous tubercles; interstices almost glabrous; ventrite 5 (Figs 13C, D) evenly covered with short adpressed setae emerging from subcordiform tubercles; lateral projection shallow.

Sternite IX (Figs 13G, H) c. 340 µm long, with moderately long anterior strut (distal end broken off in specimen figured in 13G), apical corners rounded, each with one lateroapical seta and one inconspicuous sublateroapical seta; apical margin slightly broadly emarginate; longer paraproct almost reaching apical margin.

Aedeagus (Figs 13K–N) similar to that of *Ancyronyx minutulus* (see Freitag and Jäch 2007: figs 15a, b), but distinctly larger (c. 350 μm long), relatively stouter and without long setae. Median lobe moderately long and moderately slender, with few indistinct pores, subapically straight, not widened, c. 90 μm wide, apically distinctly curved ventrad (lateral view, Figs 13M, N); tip rounded; ventral sac weakly sclerotised except for lateral rim (Figs 13K, M); fibula weakly sclerotised, inconspicuous in transillumination; corona inconspicuous. Phallobase asymmetrical, bent lateroventrad, distinctly longer ventrally, with conspicuous, strongly sclerotised margins; basolateral (penile) apophyses inconspicuous; ejaculatory duct well scleotised and conspicuous in transillumination. Parameres elongately subtriangular, rather short, reaching about basal 0.67 of aedeagus, almost contiguous ventrally, subapically slightly widened ventrad; apices with one apical and one subapical very short setae (Figs 13K, M); basal margin oblique and not conspicuously emarginate (lateral view, Figs 13M, N).

Ovipositor (Fig. 13O, P) c. 410 µm long. Stylus slender, rather staight, with various apical sensilla. Coxite moderately stout, distinctly shorter than in specimens of the *Ancyronyx patrolus* species group, but longer than in those of the *Ancyronyx variegates* group; outer margin concave; all over with several rather short and broad, peg-like spines, increasing in size and density apically at lateral margins; inner margin pubescent; basal portion short. Valvifer moderately longer than coxite; fibula slightly curved.

Secondary sexual characters: Sternite VIII in male short, weakly sclerotised and with very short median strut; in female (Fig. 13I) distinctly longer, more sclerotised than in male medially emarginate; apical corners rounded and with small seate; median portion with dense micro-pubescence. Tergite VIII in female (Fig. 13E) subtriangular, almost as long as wide (c. 210 µm long, 230 µm wide), with few moderately short setae; condyles large and conspicuous. Tergite VIII in male (Fig. 13F) subsemicircular, distinctly wider than long (c. 170 µm long, 230 µm wide), shorter than in female, reticulate; apical half with moderately short setae. Ventrite 5 in female (Fig. 13C) subtriangular (c. 230 µm long, 400 µm wide); in male (Fig. 13D) broadly oval and distinctly shorter (c. 180 µm long, 370 µm wide).

#### Adult differential diagnosis.

In its colour patterns, *Ancyronyx buhid* resembles *Ancyronyx patrolus*, *Ancyronyx punkti* and especially *Ancyronyx pseudopatrolus* from Palawan. The new species can be easily distinguished by the combination of body morphometric and genital characters (body, especially abdomen and elytra, relatively wider (CL/EW c. 2.0; EL/EW c. 1.45) than in other species; legs not distinctly longer than body; coxite of ovipositor moderately stout; aedeagus with straight main piece (not widened subapically), almost contiguous parameres ventrally, very short and few parameral setae.

#### Larval diagnosis

**(based on sixth instar).** Colour (Fig. 7) dorsally dominantly dark brown except for yellow lateral head, clypeus and labrum, most anterior portion of pronotum and the almost entire first abdominal segment; most specimens additionally with yellowish to pale brown (preterminal) abdominal segment VIII (at least posterior portion) and apex of abdominal segment IX (up to c. posterior 0.15). Legs, mouthparts, ventral head, thorax, and abdomen yellowish to pale brown, but some specimens with darker brown thoracic venter and ventral abdominal segment IX.

HW 0.31 mm; entire larva about 3.1 mm long. Body elongate, wider than that of *Ancyronyx minerva* and *Ancyronyx tamaraw*, but similar in the external characters, except for the following: posterolateral projections (Figs 7, 14A) of abdominal segments II–VI usually reaching or slightly overreaching posterior segment margins. Lateral rim of thorax and abdomen with scattered long, trichoid setae. Dorsal sagittal line slightly impressed from prothorax to abdominal segment V and without tubercles.

Head (Figs 7, 14B–E) widest posterior 0.3, not subparallel in posterior half, dorsolaterally with a pair of moderately long setae and one pair near the frontoclypeal suture (Fig. 14B); lateral setae of various size, very short to moderately long. Frontal suture inconspicuous; subbasal fringe of clypeus with rather short fasciculate setae. Ventral side (Fig. 14D) dominantly rugulose; basolateral areas and genae (inbetween setae) glabrous (Fig. 14C).

Antenna as in Figs 14D, E, c. ¼ as long as head; scape setae fasciculate; pedicel long; flagellum and sensorium subequal in length.

Labrum (Fig. 14B) with subapical fringe of ramose setae and few lateral trichoid setae. Maxilla (Fig. 14D) with parallel-sided stipes; maxillary palpus (Figs 14D, E) slightly slenderer than in *Ancyronyx tamaraw*. Labial mentum (Fig. 14D) with lateral margin slightly sinuously curved (concave in posterior half), narrowest at basal 0.2; pair of trichoid setae moderately short (reaching anterior margin), inserted sublaterally at anterior 0.2; pair of subapical lateral setae fasciculate; subbasal pair of setae ramous. Submentum short, not clearly partitioned from somewhat protruding semicircular ligula which is conspicuously covered with setiform microstructures (Fig. 14D); labial palpi as in *Ancyronyx tamaraw*.

Pro-, meso-, metathorax and legs (Figs 7, 14F) almost as in *Ancyronyx minerva*. Pronotum with rather inconspicuous small round signa (glabrous areas) in posterior half. Ventral sclerites of thorax (Fig. 14E) rugulose, not glabrous; venter of metathorax with conspicuous sagittal tuberculate ridge (similar to that of the venter in abdominal segment I).

Abdomen (Figs 7, 14A, H, I) without conspicuous dorsosagittal carina except for the anterior half of segment IX (Fig. 12G); squamose setae at posterior rim of segments I–VIII large (Figs 14A, H). Ventral sclerite of segment I with distinct sagittal ridge in anterior half (Fig. 14F), reaching c. 1/2 to 2/3 of segment length. Apex of segment IX emarginate (sometimes inconspicuous due to apical setae). Operculum (Fig. 14I) almost twice as long as wide, basal portion glabrous.

#### Variation between larval instars.

The available prefinal instar specimens vary only slightly from the description above, namely by the relatively slenderer thoracic and abdominal segments, the smaller and rather inconspicuous spiracles near the posterolateral projection, the slightly broader legs with fewer setae, and the relatively longer lateral setae on thorax and abdomen.

#### Larval differential diagnosis.

The species can most easily be distinguished from any other known*Ancyronyx* larva by the obviously pale first abdominal segment. The general shape and the proportions of the larva of this species resemble those of the *Ancyronyx patrolus* group, from which it can be additionally distinguished by the anterior yellow band, that is medially narrower (not extended as in several species of the *Ancyronyx patrolus* group) and the character combination of long sagittal crest of the first abdominal segment venter, slightly impressed dorsosagittal line without protruding tubercles. From the species of the *Ancyronyx variegatus* group, this larva can be distinguished easily by its spindle-shape habitus (subsemicircular in cross section) and the rather short posterolateral appendages.

#### Distribution.

Known only from Mindoro Island where this new species was recorded from various streams in the province of Oriental Mindoro.

#### Ecology.

Both, adult and larvae of *Ancyronyx buhid* occur in medium sized, unpolluted rivers in mountainous areas. This suggests an affinity to undisturbed habitats. The relatively highest abundances were found on submerged wood and rough rock surfaces in runs and riffles. Some root packs and partly submerged grass bunches in riffles were also found to be densely colonised with the species. Much more rarely it was found among bottom gravels in runs and calm pools, where specimens were possibly just shifted by drift.

#### Remarks.

One male specimen from site “TR2f” varies in regard to the primary and secondary sexual characters, namely the length of tergite VIII, ventrite 5 and aedeagus. Since all other characters do not differ from the type material, this is regarded as an abnormality caused during pupation.

### Ancyronyx schillhammeri

Jäch, 1994

http://species-id.net/wiki/Ancyronyx_schillhammeri

Figs 8, 9 , 15A–H

Ancyronyx schillhammeri Jäch, 1994: 617–619 (adult description), Freitag and Pangantihon 2010: 133–137 (faunistic records).

#### Material examined.

1 ♀ [FR029], 1 L (0.61 [FR030]) (ZSM) “PHIL.: Mindoro, San Teodoro, Tukuran Riv.; small lowld.riv.; riffle & run; woodlitter, gravel; sec.veget.; c. 30m asl., 13°25'34"N, 120°58'37"E 23.4.1994, leg. Freitag (303a)M’’; 3 ♂♂, 1 ♀ (PNM) “PHIL.: Mindoro Oriental, Bongabong, Brgy. Formon, Pastuhan, Tangisan Falls; deep mountain valley, sec. forest, submerged wood, riffle, c. 200 m asl., c. 12°43'N, 121°23'E; 27.10.2011 leg. Freitag (318a)M”; 3 ♂♂ (CFM) “PHIL: Or. Mindoro, Roxas, Bagumbayan, polluted Magugo River; sec.veget.; submerges wood, run; 6m asl., c. 12°35'27"N, 121°31'00"E; 05.6.2000 leg. Freitag & Pangantihon (329c)M”; 1 ♂, 4 ♀♀ (CFM) “PHIL: Or. Mindoro, Roxas, Brgy. San Vicente, Baroc River; subm. wood; gravel flood plains; c. 12°37'07"N, 121°24'06"E, 90m asl; leg. Freitag 1 Apr.2013 (BRf)M”; 4 ♂♂, 7 ♀♀ (CFM) “PHIL: Or. Mindoro, Roxas, Brgy. San Vicente proper, Taugad River; subm. wood; sec.veget.; c. 12°37'06"N, 121°23'49"E, 100m asl; leg. Freitag 2 Apr.2013 (TR1f)M”; 5 ♂♂, 1 ♀ (SMTD, ZSM) “PHIL: Or. Mindoro, Roxas, Brgy. San Vicente, Tauga River; subm. wood, run; sec.veget.; c. 12°37'18"N, 121°22'58"E, c. 140m asl; leg. Freitag 28.Nov.2011 (TR2f)M”; 1 ♂, 3 ♀♀ (ZMUC) “PHIL: Or. Mindoro, Roxas, Brgy. San Vicente, Sitio Tauga Diit, Baroc River tributary Tauga Daka; subm. wood in run; sec.veget.; c. 12°38'05"N, 121°19'33"E, c. 530m asl; leg. Pangantihon, 23 Jan. 2013 (TDR3f)M”; 1 ♀ (CFM) “PHIL: Or. Mindoro, Roxas, Brgy. San Vicente, Sitio Quirao, Baroc River tributary Hinundugan River; subm. wood; c. 12°36'23"N, 121°23'29"E, c. 118m asl; leg. Pangantihon, 22 Jan.2013 (HR1f)M”; 1 ♀ (CFM) “PHIL: Oc. Mindoro, San Jose, Brgy. “Central” Purok Tunnel, Busuanga River; rural, cogon gras roots, riffle & run, c. 73 m asl., c. 12°27'51"N, 121°02'08"E; 07.VI.2012 leg. Freitag & Pangantihon (330b)M”.

#### Larval diagnosis

**(based on a single presumably sixth instar specimen).** Colour in last instar larva predominantly brown as in Fig. 9; dorsal head darkest to almost black at pronotal disc; lateral head, antennae, anterior and lateral pronotal margins, legs (except for tip of claw), lateral abdominal segment margins and conical projections, posterior abdominal tip and areas around the sagittal line (especially thoracic area) distinctly paler, yellowish to pale brown. Ventral side entirely pale except for pale brown gula, maxillae and labium; ventral part of genae conspicuously dark brown.

HW c. 0.60 mm; entirely c. 3.8 mm long.

**Figure 15.**
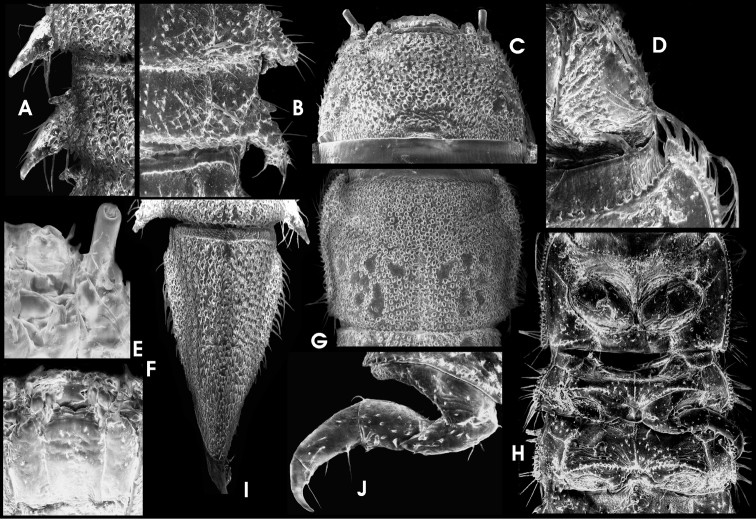
*Ancyronyx schillhammeri* Jäch, 1994, larva (SEM photographs) not to scale): **A** detail of abdominal segments II–III, dorsal, with posterolateral projections and spiracles **B** detail of abdominal segments VI–VII, ventral, with posterolateral projections and spiracles **C** head, dorsal **D** detail of head and prothorax, ventral **E** antenna, ventral **F** maxillae and labium, ventral **G** pronotum with signa, dorsal **H** thorax, ventral **I** abdominal segment IX, dorsal **J** midleg, ventral.

Body shape of the *Ancyronyx variegatus* group type, generally very similar to that of *Ancyronyx procerus* (comp. Freitag and Balke 2011: 72–75) in the external habitus, except for the following: Posterolateral projections (Figs 9, 15A, B) of abdominal segments IV–VIII slightly broader and stouter; spiracles distinctly larger, very prominent; entire lateral margin with distinct long trichoid setae; tubercles much more prominent (especially at dorsal side).

Head (Figs 15C–F) distinctly widest basally, slightly conical anteriad; without median pointed projection at frons; the pair of sublateral anterior projections between antenna and clypeus rather shallow (Fig. 15C). Frons moderately densely and equally covered with prominent setiferous tubercles; setae very short. Genae rugose, with irregular depressions, ridges, and scattered tubercles (Fig. 15D); lateral glabrous area with stemmata elongately subtriangular (Fig. 15D). Antenna (Fig. 15E) less than half as long as head. Scape short, slightly longer than wide, with subapical fringe of stout sensilla; pedicel cylindrical less than three times as long as scape and c. three times as long as wide, with few apical sensilla; flagellum and sensorium as in *Ancyronyx procerus*. Ventral side (Fig. 15F) with well-developed longitudinal crests bordering the stipes. Labrum broad, c. 3.5 times as wide as long; lateroapical edges rounded; entire visible dorsal surface with small setiferous tubercles. Maxilla (Fig. 15E, F) almost as in *Ancyronyx procerus*. Labium (Figs 15E, F) with mentum widest in apical half; pair of moderately long trichoid setae inserted sublaterally at anterior 0.3; some additional trichoid setae present at lateral margin in apical half; pair of apicolateral teeth inserted at a distinct subapical crenation; submentum straight, without conspicuous median ridge, apically broadly concave.

Prothorax (Fig. 15G, H) slightly wider than long; tergum with irregularly shaped and round signa in posterior half; median and submedian pairs clearly defined by bordering tubercles, not fused (Fig. 15G). Venter of prothorax (Fig. 15H) similar to that in *Ancyronyx procerus*, but anterior sclerites distinctly shorter, oblique, not subtriangular; anterior margin with conspicuous setiferous tubercles; anterior and lateral sclerites partly fused near anterior prothorax margin; transverse sutures dividing the lateral sclerites distinctly ending before lateral margin. Venter of meso- and metathorax (Fig. 15H) with more conspicuous setiferous tubercles particularly at posterior margins of anterior sclerites.

Legs (Figs 15H, J) proportioned as in *Ancyronyx procerus*, but tubercles and setae larger and more distinct. Subbasal tooth of claws long and trichoid, overreaching tip of claw.

Abdominal terga (Figs 9, 15A, B, I) with slightly depressed groove along sagittal line at least from 1^st^ up to 4^th^ segment; posterior terga margins with rim of squamose setae. Posterior venter margins with rim of trichoid setae. Segment IX (Fig. 15I) dorsally with shallow sagittal crest formed by densely arranged tubercles bearing large trichoid setae; apex widely rounded to slightly truncate; ventral side rugose, not glabrous. Operculum without longitudinal ridges, entirely rugose and covered with conspicuous scattered setae.

#### Larval differential diagnosis.

The larvae of *Ancyronyx schillhammeri* are easily distinguishable from all other known *Ancyronyx* larvae of Mindoro by their larger size, the somewhat dorsoventrally depressed habitus, the much larger and protruding posterolateral appendages, as typical for the *Ancyronyx variegates* species group. Among this species group, it resembles the larva of *Ancyronyx procerus* in colour, but can be clearly distinguished by the absence of the pointed projections at median frons, the more shallow projections between antenna and clypeus, the larger and more protruding spiracles, the conical head shape, and the surfaces of head, thorax, and abdomen that are densely covered with larger tubercles bearing long conspicuous setae. From *Ancyronyx helgeschneideri* itis easily distinguishable by the darker colour, the pale dorsosagittal stripe, the dark dorsal abdominal segment IX and the broader and conical head.

#### Distribution.

Only known from Oriental Mindoro and one locality of Occidental Mindoro near San Jose.

#### Ecology.

*Ancyronyx schillhammeri* occurs exclusively on submerged wood. Decaying pale light woods appear to be preferred by the species. At the sites of the Baroc River catchment, which were sampled regularly throughout the year, the abundance of this species was found to increase distinctly towards the end of the dry season (February to April) and declines rapidly with the beginning rainy season, presumably due to wash out. It is found in both habitat types: clean, cool and torrent rhithral creeks and rivers as well as warm, mesosaprobic lowland streams. This suggests less specific ecological requirements in terms of stream hydraulics, water temperature, and water quality.

**Figure 16.**
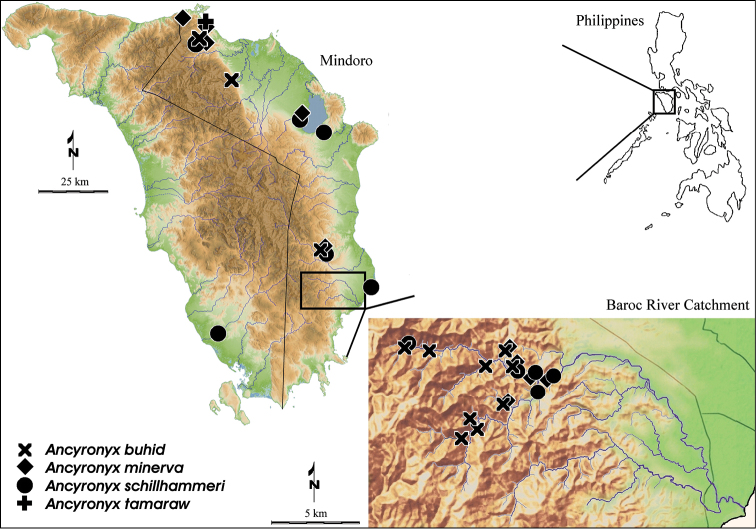
Collecting sites of the *Ancyronyx* species in Mindoro Island, including enlarged map of the Baroc River Catchment.

### Key to the adult *Ancyronyx* species of Mindoro

**Table:** 

1	Elytra with yellowish “X”-shaped colour pattern (Fig. 8); combined length of pronotum and elytra (CL) ≥ 1.8 mm; legs very long (≥ 1.2 times of body length)	*Ancyronyx schillhammeri*
–	Elytra with four isolated yellowish colour patches; CL ≤ 1.4 mm; legs long to moderately long (≤ 1.1 times of body length)	2
2	Body about half as wide as long (CL/EW c. 2.0), elytral shoulder distinctly wider than pronotum; posterior yellowish elytral patches more or less round, not distinctly elongate	*Ancyronyx buhid*
–	Body less than half as wide as long (CL/EW ≥ 2.2), elytral shoulder about as wide as pronotum; posterior yellowish elytral patches elongate, distinctly longer than wide	(*Ancyronyx minerva* species subgroup) 3
3	Pronotum anteriorly with transverse white to yellowish band; femora entirely dark coloured; anterior yellowish elytral patches large, extending over more than 3/4^th^ of elytral width (Fig. 2)	*Ancyronyx minerva*
–	Pronotum entirely dark, without transverse band; femora predominantly yellowish coloured; anterior yellowish elytral patches small, extending over ½ or less of elytral width (Fig. 3)	*Ancyronyx tamaraw*

### Key to the larvae of *Ancyronyx* species of Mindoro

**Table:** 

1	Body flattened dorsoventrally (depressed), only slightly vaulted. Posterolateral abdominal projections large, conical, with tip posterolaterad directed. Dorsal colour predominantly dark brown, with distinct pale sagittal stripe in anterior half (Fig. 9)	*Ancyronyx schillhammeri*
–	Body spindle-shaped, elongate, subsemicircular in cross section. Posterolateral abdominal projections small, lobate, with tip posteriad directed. Predominant dorsal colour brown, with transverse pale yellowish bands or dots, without any pale longitudinal stripe (Figs 5–7)	2
2	First abdominal segment almost entirely yellowish, appearing as an obvious pale band. Dark portion of the pronotum medially extended anteriad, yellowish anterior pronotal band medially narrower. (Fig. 7)	*Ancyronyx buhid*
–	First abdominal segment almost entirely brownish dark, no obvious pale band present at the dorsal abdomen. Anterior pronotal yellowish band regularly shaped or extending medially, not narrower along the midline (Figs 5–6)	(*Ancyronyx minerva* species subgroup) 3
3	Anteriomedian portion of the dorsal head yellowish pale, surrounded by dark areas. Last abdominal segment c. 2.3 times as long as wide, with pale apical and median areas and distinctly dark portion inbetween (Fig. 6)	*Ancyronyx tamaraw*
–	Entire dorsal head disc brownish dark. Last abdominal segment c. 2.2 times as long as wide, any pale dorsal colour pattern lacking or limited to tip (Fig. 5)	*Ancyronyx minerva*

### Updated check list of the Philippine species of Ancyronyx

1. *Ancyronyx buhid* Freitag, 2013 (Mindoro)
2. *Ancyronyx helgeschneideri* Freitag & Jäch, 2007 (Palawan, Busunga)
3. *Ancyronyx minerva* Freitag & Jäch, 2007 (Palawan, Mindoro)
4. *Ancyronyx minutulus* Freitag & Jäch, 2007 (Palawan)
5. *Ancyronyx montanus* Freitag & Balke, 2011 (Palawan)
6. *Ancyronyx patrolus* Freitag & Jäch, 2007 (Palawan, Busuanga)
7. *Ancyronyx procerus* Jäch, 1994 (Busuanga, Borneo, Vietnam)
8. *Ancyronyx pseudopatrolus* Freitag & Jäch, 2007 (Palawan)
9. *Ancyronyx punkti* Freitag & Jäch, 2007 (Palawan)
10. *Ancyronyx schillhammeri* Jäch, 1994 (Mindoro)
11. *Ancyronyx sophiemarie* Jäch, 2004 (Sibuyan)
12. *Ancyronyx tamaraw* Freitag, 2013 (Mindoro)

## Discussion

During the last two decades, the Philippine Islands have received increasing attention in biodiversity research, not least because they are classified as a major biodiversity hotspot in global scale (Myers et al. 2000). However, for several taxa including Elmidae and other freshwater macroinvertebrates, it still requires substantial efforts to record and to describe the majority of species and their distribution.

Four species of *Ancyronyx* are now recognised and formally described from Mindoro Island based on the study of a copious collection of museum specimens and the material retrieved from a comprehensive survey of the Baroc River Catchment in southern Mindoro. Only one of them, *Ancyronyx minerva*, is recorded beyond Mindoro. *Ancyronyx schillhammeri* and *Ancyronyx buhid* appear to be endemic to the island. The high rate of island endemism reflects the biogeographic history of the island. Mindoro is a remnant of a fragment of the Eurasian continental margin and is not part of the Luzon arc of islands of marine volcanic origin (Hall 1998). Despite its recent close vicinity to Luzon, the two islands remained largely isolated in the Quaternary, even during Pleistocene when low sea levels have formed land bridge interconnections of several Philippine islands, but presumably Greater Palawan, Mindoro, and Greater Luzon remained separated based on Pleistocene sea-level low stands represented by the 120 m isobath (Sathiamurthy and Voris 2006).

Therefore, it requires more in depth phylogenetic and biogeographic studies to explane the distribution of *Ancyronyx minerva* at both sides of the Mindoro Strait.

The phylogenetic relationship of *Ancyronyx buhid* with other members of the genus is still ambiguous. Several taxa (probably new species) from the Philippines and Sulawesi that resemble *Ancyronyx buhid* still await their description (unpublished material of the author and at NMW). After this material has undergone detailed study and molecular genetic analysis sound conclusions might be drawn.

The fact that *Ancyronyx* (and very most other Elmidae) live permanently under water and respire by a microplastron (Kodada and Jäch 2005) makes them sensitive to water pollution. The vivid and specific colour patterns of adult *Ancyronyx* species enabling an easy identification, as well as the availability of regional identification keys for both, larvae and adults, allow their potential use as bioindicators. Among the species of Mindoro, *Ancyronyx schillhammeri* was recorded from clean to moderately polluted streams making it unsuitable as a bioindicator. The remaining Mindoro species seem to be ecologically adapted to clean and rather undisturbed waters. However, *Ancyronyx tamaraw* is too rare to serve as good bioindicators and *Ancyronyx minerva* is occasionally detected in slightly polluted streams in low abundances (Freitag and Pangantihon 2010), suggesting a low indicator strength. Therefore, *Ancyronyx buhid* in particular has the highest potential value to be used as saprobic indicator. Its frequent occurrence in suitable habitats and the easy identification by the distinguishing elytral colour pattern and broad elytral shoulders in adults, as well as the unique yellow abdominal pattern in larvae, make it a suitable tool for biomontoring, even for non-entomologists. However, ecological evaluations of larger scale are needed to confirm these preliminary findings.

## Supplementary Material

XML Treatment for Ancyronyx minerva

XML Treatment for Ancyronyx tamaraw

XML Treatment for Ancyronyx buhid

XML Treatment for Ancyronyx schillhammeri
